# Comparison of GHG emissions from annual crops in rotation on drained temperate agricultural peatland with production of reed canary grass in paludiculture using an LCA approach

**DOI:** 10.1016/j.heliyon.2023.e17320

**Published:** 2023-06-15

**Authors:** Henrik Thers, Marie Trydeman Knudsen, Poul Erik Lærke

**Affiliations:** Department of Agroecology, University of Aarhus, Blichers allé 20, DK-8830, Tjele, Denmark

**Keywords:** Bog, CH_4_, Fen, N_2_O, RCG, Soil emissions, Wetlands

## Abstract

Drained peat soils contribute significantly to global human-caused CO_2_ emissions and reducing peat degradation via rewetting is high on the political agenda. Ceasing agricultural activities on rewetted soils might lead to land owner resistance and high societal expenses to compensate farmers. Continued biomass production adapted for wet conditions on peat soils potentially minimizes these costs and helps supplying the growing demand for e.g. materials, fuels and feed. Here we used a life cycle assessment approach (cradle to farm gate) to investigate the greenhouse gas (GHG) emissions related to three cases by applying IPCC (Intergovernmental Panel on Climate Change) emission factors and specific site conditions at a bog and a fen site that represent widely distributed temperate peat soils. Besides soil emissions, upstream emissions from input, operational emissions and emission related to rewetting construction work were included. The analyzed systems were deeply drained cash cropping on agricultural bog (potatoes (*Solanum tuberosum* L.), spring barley (*Hordeum vulgare* L.) and oat (*Avena sativa* L.), permanent Reed canary grass (RCG) (*Phalaris arundinacea* L.) production on non-drained bog and permanent RCG production on shallow-drained fen. The annual mean water table depths (WTD) were −70, −38 (estimated) and −13 cm, respectively. Results showed estimated GHG emissions of 40.5, 26.1 and 20.6 Mg CO_2_eq ha^−1^, respectively, corresponding to a 35% GHG reduction for the non-drained bog case as compared to the drained bog case, despite that the obtained WTD due to ceased drainage did not adhere to the IPCC rewetting threshold of −30 cm. Emissions related to crop management represented 7, 14 and 19% of total emissions. In the RCG cultivation on fen case, the WTD were controlled primarily by the water table of the nearby stream and total GHG emissions were even lower as compared to the RCG production on the non-drained bog reflecting the difference in WTD. Rewetting projects need to include careful knowledge of the specific peat area to foresee the actual reduction potential.

## 1 Introduction

Peat soils are characterized by high carbon (C) content, holding 25% of total global soil C, while only covering 3% of the terrestrial land worldwide [1]. Agricultural activities on peatland depend on drainage, which leads to increased peat oxidation due to increased oxygen supply, as a consequence of lowered water table [2,3]. Thus, the vast C stock held in the peat soils are emitted to the atmosphere [4] at peat subsidence rates that has previously been reported to be in the range from 0.08 to 2.2 cm year^−1^ [5]. In addition to the CO_2_ emissions from soil degradation come other sources of C losses and greenhouse gasses (GHGs), namely dissolved organic C (DOC), methane (CH_4_) and nitrous oxide (N_2_O) [2,6]. Whereas CH_4_ emissions from drained peat soil are minor, the levels of DOC and N_2_O can be considerable, although also representing the most uncertain quantities [6]. Despite general low CH_4_ emissions from drained soils, potential significant CH_4_ emissions from drainage ditches need to be included in the calculation [6,7].

Like CO_2_ emissions, also the two GHG species CH_4_ and N_2_O are influenced by the water table through the oxygen availability and resulting peat degradation, N mineralization and redox potential while DOC losses depend also on water flows [1,6]. However, the relationship between the different GHG species and water table level varies [6,8]. For instance, CH_4_ emissions decrease with increasing water table depth (WTD; negative values indicate a mean WTD below the peat/soil surface) since an upper unsaturated zone of the soil profile secures oxidation of the major part of CH_4_ produced in the deeper water-saturated zone [2]. Contrary to the CH_4_, N_2_O emissions increase with WTD due to a combined effect of increased mineralized N and less complete denitrification to N_2_ [3,9]. Total soil derived GHG emissions has been suggested to be lowest when WTD range between −40 and −20 cm by Hatano (2019) [1]; −13 and −5 cm by Evans et al. (2021) [10] and at −5 cm by Jungkunst et al. (2008) [8].

Natural peat soils are soil C sinks by definition, since they have been building up large C stocks, typically over millennia [1]. The slow organic matter decomposition in pristine peat soils is a consequence of water-saturated conditions, which potentially entail high CH_4_ emissions due to the low redox potential [2]. The potentially high CH_4_ emissions and the possible difficulties in reestablishing vegetation and the derived resumed C sequestration, e.g. sphagnum mosses, may challenge the overall peat soil status as GHG sink on rewetted sites, i.e. previously drained soils where drainage is ceased [11,12]. Nevertheless, pristine peat soils have shown to be C sinks even when all GHGs are taken into consideration, which is eventually also expected to be the case for rewetted sites, provided establishment of a successful peat-forming vegetation, although a timescale for this of half a century has been suggested, due to possible long term consequences of the previous drainage and farm management conditions [2,11]. Therefore, rewetting has been suggested as a management action to turn soil C degradation into C uptake [2,4].

In order to maintain the crop production potential of peat soils while using rewetting or shallow-drainage as a GHG mitigating management, the concept of paludiculture has been introduced as an alternative to farming depending on deep drainage [4,13]. Reed canary grass (*Phalaris arundinacea* L.) (RCG) has proved a well-suited crop for paludiculture with high yield potentials when cultivated on poorly drained peat soils [[14], [15], [16]]. In addition, it was found suited for protein extraction in a biorefinery process [17] as well as for production of bioenergy [18], and thus the economic performance is accounted for with a possible commercial application of the output.

An important factor determining GHG levels from peat soils is the basic distinguishing between fens and bogs dependent on the dominant water source and resulting nutrient status [2,6]. Fens are classified as minerotrophic soils, being mainly water supplied by ground and surface water sources, which often transport nutrients from elevated neighboring agricultural areas [19], typically leading to relatively nutrient rich peat soils [20]. Raised bogs are water supplied by precipitation leading to nutrient poor ecosystems dominated by sphagnum mosses and low pH, and classified as ombrotrophic soils [19,20]. Due to the very different nutrient properties of these two peatland types, reported GHG emissions are in many cases disaggregated between nutrient poor and rich peat soils [21]. However, in the case of e.g. Denmark the same emission factor for both fens and bogs is applied [22], although these emission factors (EFs) are on the Tier 2 level, that should generally reflect a more accurate and targeted EF in the IPCC emission system as compared to the overall Tier 1 level. Effects of using disaggregated EFs for assessment of the climate effect of paludiculture has not been reported before.

As paludiculture and ceased drainage affect total system related GHGs in complex ways related to changed water table depth (WTD), specific peat properties and various management related operations (fertilization, field operations, transport, etc.) [2,9,23], the overall carbon footprint should be analyzed by a life cycle assessment approach in order to get a more complete picture [24]. The complexity is stressed by the number of possible combinations of management, peat soils, climate zones and drainage regimes that can be analyzed, which again complicates optimal solutions for decision makers. Järveoja et al. (2013) [25] made a comparison of different after-use of a peat extraction site in Estonia. Although this analysis included two reed canary grass cases and one rewetted case, the combination (paludiculture) was not included, and the need of more knowledge on the subject has been acknowledged [2]. Lahtinen et al. (2022) [26] used an LCA approach to evaluate two paludiculture derived products and stressed that more research was needed particularly on the biomass cultivation part of the full product life-cycle. Thus, in order to pave the road for future peat soil management, additional investigations of possible outcomes of peat soil cultivation management and associated potential GHG reductions are needed. Due to the currently unclear definition of paludiculture, we defined the present cases of paludiculture as *crop production on peatland with shallow WTD*, which thus includes a wider range of WTDs than the threshold WTD of −30 cm for rewetted sites [21].

The aim of this study was to apply an LCA approach to two specific Danish peat site conditions to represent three cases of temperate peatland management and to sum up the total carbon footprint by applying IPCC peat soil associated GHG emission factors, emissions related to cultivation and construction work as well as upstream emissions. Hereby this study becomes an example of how to assess current and future GHG emissions from peat lands where actual measurements are not available, which is often the case for minor sites. The three cases include traditional crop rotation on an agricultural raised bog, paludiculture RCG production after drainage blocking on the same raised bog, and likewise paludiculture RCG production on a shallow-drained fen peatland. To our knowledge such comparison of paludiculture on bog and fen peatland has not previously been conducted. We hypothesized that the paludiculture RCG cases would show considerably lower GHG emissions as compared to the conventional drained agricultural farming system.

## 2 Material and methods

### 2.1 Aim and scope

**Fig. 1:**
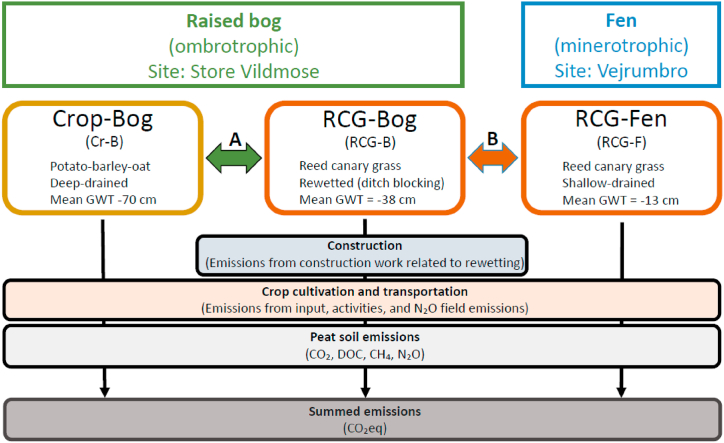
Schematic overview of the three cases treated in this study and the included emission sources for each case. The capital A and B mark the two comparisons made in this study. A: Comparison of present crop production on deep-drained bog and reed canary grass production on bog subsequent to drainage blocking. B: Comparison of likewise reed canary grass production on bog and fen.

The overall aim of this study was to assess the GHG reduction potential when changing crop production on peatland from drained to non-drained and wet soil conditions in the temperate climate zone. This was done by i), comparing GHG emissions from conventional drained agricultural farming of potatoes (*Solanum tuberosum* L.), spring barley (*Hordeum vulgare* L.) and oat (*Avena sativa* L.), to paludiculture of permanent reed canary grass (RCG; *Phalaris arundinacea* L.) on a non-drained ombrotrophic peat soil (Fig. 1). ii) comparing GHG emissions from RCG paludiculture production on rewetted ombrotrophic peat soil to RCG paludiculture production on shallow-drained minerotrophic peat soil using disaggregated IPCC Tier 1 emissions factors (Fig. 1). System borders are cradle to farm gate, meaning that it includes the cultivation steps and upstream emissions from input and that all harvested biomass is transported to the farmyard and no further (farm gate boundary). Covered GHG emissions in the analysis are CO_2_ (including CO_2_ from DOC), CH_4_ and N_2_O and the global warming factors of 25 for CH_4_ and 298 for N_2_O in a 100-year time perspective were applied, currently used for conversion to CO_2_ equivalents (CO_2_eq) in Denmark's National Inventory Reports under the United Nations Framework Convention on Climate Change and the Kyoto Protocol [27]. The functional unit is 1 ha of peat soil, and in addition the results are presented using 1 kg harvested biomass dry matter (DM) and 1 kg harvested crude protein as functional units, because protein extraction is a possible application of RCG [17]. Comparisons are performed by summing up CO_2_eq for one year. Finally, sensitivity analyses are conducted in order to clarify the impact of uncertainty on applied peat soil emissions factors and biomass yields.

### 2.2 Data sources and peatland sites

**Fig. 2:**
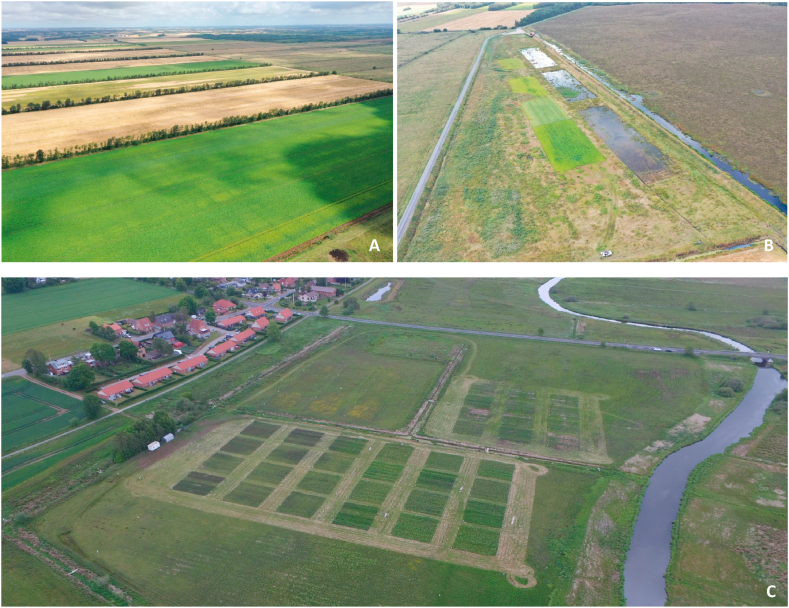
The sites considered in this study as representative sites for bog and fen peat soils in the moist temperate zone. A: Current cash crop cultivation in the deep-drained raised bog of St. Vildmose. B: An experimental site of St. Vildmose, where drains are blocked, used for perennial crop research, located between a natural part of the bog to the right and the deep-drained crop production to the left of the paved road. C: The shallow-drained fen site near Vejrumbro.

The applied assumptions on crop management, field operations and yields are derived partly from literature values and partly from experiments and experience from two specific sites, namely Store Vildmose (57° 13′ 26″ N; 9° 46′ 55″ E), which is a raised bog (Fig. 2A and B) and Vejrumbro (56°26′15.3″N, 9°32′44.1″E), representing a typical river valley fen (Fig. 2C). The Vejrumbro fen site management during the last 20 years was a mixture of grazing by heifers and grass cutting. The grass pasture was renewed every fifth year until 2018, when it became a permanent grass experimental site, and also maintenance of open drains ceased 2018. WTD for the fen are largely controlled by water levels in the nearby stream and average WTD had been recorded to average approximately −30 cm at summertime and 0 cm at winter time in the years as an experimental site.

Data on WTD, biomass yield and plant N content were derived from the two sites. IPCC Tier 1 default peat soil emission factors were applied (Table 1) in order to secure the representativeness of RCG production on bog and fen peat soils in the moist temperate zone.

**Table 1:** Inventory table for GHG emissions, yields and input used for calculations of the carbon footprint in the main and sensitivity analyses of the three cases investigated. *Main* indicates the values applied in the main analysis with lower and upper values applied in the sensitivity analysis of uncertainty on specific parameters. Lower and upper values for diesel as well as direct and indirect N_2_O are associated to the sensitivity analyses on yields. Cr–B is the cash-crop on bog case, RCG-B, is the reed canary grass on bog case and RCG-F is the reed canary grass on fen case. Regarding the Cr–B scenario, the Field emissions, Input and Output are expressing an average of the three crops in the crop rotation (i.e. 1/3 ha of potatoes, 1/3 ha of spring barley and 1/3 ha of oat), see Table A3 and A4 for further details. All values are per ha per year.

Case	Cr-B			RCG-B			RCG-F		
**Emission**	**Main**	Lower	Upper	**Main**	Lower	Upper	**Main**	Lower	Upper
***Peat soil emissions***									
CO_2_ (Mg C)	7.9^a^	6.5^a^	9.4^a^	5.3^b^	3.7^b^	6.9^b^	3.6^c^	1.8^c^	5.4^c^
DOC∗ (Mg C)	0.31^d^	-	-	0.24^d^	-	-	0.31^d^	-	-
CH_4_# (kg C)	43.7^e^	10.5^e^	76.9^e^	1.4^b^	0.5^b^	2.2^b^	49.0^c^	8.5^c^	89.6^c^
N_2_O (kg N)	13.0^e^	8.2^e^	18.0^e^	4.3^b^	1.9^b^	6.8^b^	1.6^c^	0.6^c^	2.7^c^
***Field emissions***@									
N_2_O, direct (kg N)	2.0	1.9	2.1	2.0	2.0	2.0	2.0	2.0	2.0
N_2_O, indirect (kg N)	1.7	1.9	1.4	1.3	2.2	0.4	1.7	2.8	0.6
Input**									
Diesel (L)	75.6	-	-	164	112	217	164	112	217
Lime (kg)	125			0	-	-	0	-	-
Pesticide (active ingredient; kg)	2.5	-	-	0	-	-	0	-	-
N (kg)	93	-	-	200	-	-	200	-	-
P (kg)^i^	22	-	-	43	-	-	43	-	-
K (kg)^i^	95	-	-	214	-	-	214	-	-
Output**									
Yield DM (Mg)	6.48	5.51^f^	7.45^f^	12.0	8.0^g^	16.0^h^	12.0	8.0^g^	16.0^h^
Yield N (kg)	118	100	136	240	160	320	300	200	400

Hyphens indicate that the specific parameter is not relevant for sensitivity analysis.

∗ Listed values are the CO_2_ emissions resulting from the dissolved organic C (DOC), which is 90% of total DOC[21].

# Includes emissions from ditches in the CR-B and RCG-F cases (Table A1 and A2), whereas there are no contribution from ditches in the RCG-B case.

@ Direct and indirect N_2_O emissions from the crop rotation/RCG cultivation is based on the N balance (Table A4 and A5).

∗∗ See Table A3 for details on the three crops in the Cr-B case.

a CO_2_ emission factor for cropland on temperate organic soil[21].

b Disaggregated value for temperate grassland, drained (no disaggregation on deep- and shallow-drained), nutrient-poor; lower and upper values are 95% confidence interval extremes[21].

c Disaggregated value for temperate grassland, shallow-drained, nutrient-rich; lower and upper values are 95% confidence interval extremes[21].

d Temperate organic drained (Cr-B and RCG-F) and undrained (RCG-B) soil[6].

e Disaggregated values for temperate drained peatland under cropping management; lower and upper values are 95% confidence interval extremes[21].

f Lower and upper yield for the Cr-B case were assumed to be +/- 15%.

g [16].

h [23] and [17].

i N, P and K fertilizer amounts were applied as NPK 14-3-15 (Yara, Norway).

### 2.3 Case details

Three cases are considered of which the first, Crop-Bog (Cr–B), includes the current cash crop rotation on an agricultural raised bog (Fig. 1, Fig. 2A). The second case, RCG-Bog (RCG-B) regards cultivation of RCG after predicted water table-rise (due to ditch blocking and dike construction) in the same raised bog as the Cr–B case [28]. The third case, RCG-Fen (RCG-F), regards corresponding cultivation of RCG on a fen site (Fig. 2C).

The cash crop farm system at the bog peat soil (Cr–B case) represents a crop rotation currently practiced on this bog peatland including field operations (e.g. ploughing) and application of pesticides and mineral fertilizer. The crops; potatoes, spring barley and oat, are aimed for human consumption and feed. To express an average year, 1/3 ha of each crop (Table 1) is considered. For determination of crop residue turnover for direct N_2_O calculation, the crop residues from a cover crop (Oil Radish; *Raphanus sativus* var. *oleiformis* Pers.) was also considered, which according to farmers practice was cultivated on 60% of the area subsequent to crop harvest. The cover crop residues were assumed to contain 46 kg N ha^−1^ (based on national experiments; e.g. SEGES (2019) [29] when incorporated into the soil, which averaged 28 kg N ha^−1^ (when considering 60% coverage). The Cr–B case is classified as deep-drained with a mean WTD of −70 cm [21,30].

The RCG cultivation of the RCG-B and RCG-F cases represents the paludiculture RGC crop when well-established, securing that the analysis is considering an average year of production. The mean annual WTDs of −38 cm and −13 cm, respectively. The WTD of −38 cm for the bog was derived from a report that predicted future WTD in a projected rewetting scenario in St. Vildmose [28], whereas the mean WTD of the fen site was measured throughout the year of RCG production in Vejrumbro [17]. Both RCG cases were applied 200 kg N ha^−1^ annually (two splits) in mineral fertilizer and no pesticide application. Diesel consumption for initial soil tillage and crop establishment was divided by 20 years, since this was assumed necessary only once, while diesel for supplementary seeding every fifth year in the established crop (without tillage) was also accounted for. A two-cut strategy was chosen for the RGC, since this strategy has been shown to optimize yields as compared to one and three to five cuts annually [17]. In the harvest process, the grass is moved followed by a forage harvester, both suited for wet conditions.

### 2.4 Yields

Crop yields in the Cr–B case were based on farmer’s information. RCG yields were based on [14,15,31] as well as new results from RCG cultivation on the experimental site of St. Vildmose (unpublished). Despite the assumed equal DM yield on the two RCG sites (Table 1), the N yield was differentiated. The N yield for the fen (25 g N kg^−1^ DM) was derived as an approximate average from Kandel et al. (2020) and Nielsen et al. (2021) [14,17] whereas N yield for the bog (20 g N kg^−1^ DM) was derived from Mander et al. (2012) [16] combined with unpublished results from the demonstration experiment shown at Fig. 2B in the St. Vildmose bog site (mean of 18.4 g N Kg^−1^ DM).

### 2.5 Peat soil and field GHG emissions

GHG emissions and C losses (CO_2_ from soil degradation, dissolved organic Carbon (DOC), CH_4_, and N_2_O) from the peat soils were based on IPCC emissions factors and if possible, disaggregated values were applied, i.e. according to climate zone, nutrient status and WTD [21] (Table 1).

Direct N_2_O–N emissions were calculated using the default EF associated with applied N and crop residue turnover N of 1% [32]. Crop residue N turnover was accounted for by applying IPCC standard values relative to crop yield [33]. Nitrate leaching from the cases was estimated by tentative N balances that included N input sources in terms of fertilizer, soil mineralization and N deposition, and N losses in terms of harvested biomass N content, NH_3_ evaporation, direct N_2_O and N_2_. Determination of mineralized N was based on the soil C degradation and C:N ratios of 26.5 for bog cases (Cr–B, RCG-B), derived from Kandel et al. (2018) and Petersen et al. (2012) [34,35] and 14.3 for the fen case (RCG-F) [17]. N deposition was assumed to be 14 kg N ha^−1^ [36]. NH_3_ evaporation was set as 5% of applied inorganic N fertilizer and on top of that 2 and 0.5 kg N ha^−1^ from annual crops and grasses, respectively [22]. For the proportion of N_2_O–N to N_2_, a ratio of 1:9 was applied which reflects wet, heavy, fertile soil in the SimDen model [37]. Nitrate leaching was assumed to be the balance surplus of which an EF of 1.1% was applied for indirect N_2_O, whereas a EF of 1% was applied for NH_3_–N [33].

### 2.6 Other emissions

Production related GHG emissions from the production of N, P and K fertilizers were assumed to be 6.6, 3.6 and 0.7 kg CO_2_eq kg^−1^, which include transport emissions [38]. Lime was assumed to have a direct emission (in CO_2_–C) of 12% of applied CaCO_3_ [32] and an additional emission of 19 g CO_2_eq kg^−1^ lime for excavation and transportation [39].

Included diesel consumption is presented per case and per crop for the Cr–B case (Table 1, Table A4). The calculated amount of diesel used for field operations was multiplied with 1.1 in order to account for the increased resistance when driving in the peat soil [40] and by 1.007 to account for lubrication oil [41]. A distance of 5 km from field to farm is included in calculation of diesel consumption for crop transportation.

### 2.7 Construction work associated with bog peatland rewetting

In St. Vildmose, the RCG-B case is assumed to be on currently (deeply) drained fields and thus includes a rewetting event. The rewetting-associated construction work should be accounted for in the RCG case and necessary resources were based on a report analyzing a potential future rewetting project in St. Vildmose covering 345 ha. The construction work includes filling up all drainage ditches and establishing an 8.5 km dike (with a membrane) along three sides of the area. 2080 h for excavators, 1640 h for bull dozers and 1110 h for dump trucks were assumed (Personal communication to Martin Nissen Nørgård, Danish Nature Agency). Diesel consumption for the machines were assumed to be 15, 24 and 10 L h^−1^, respectively. 18,600 m^2^ of membrane (1.13 mm thick) having an emissions (from fabrication) of 2.85 kg CO_2_eq kg^−1^ membrane (assuming 1424 g m^−2^) was included. Summed emissions were divided by 345 ha and 20 years in order to convert the construction emission burden to the unit kg CO_2_eq ha^−1^ year^−1^.

### 2.8 Sensitivity analysis

For the main analysis, the *main* values provided in Table 1 were applied. A number of alternative calculations have been conducted in order to investigate the consequences if these values reach the potential extremes, provided as lower and upper values in Table 1. Sensitivity analysis 1, 2 and 3 (Sa1, Sa2 and Sa3) were performed on the three most important peat soil emissions individually (i.e. CO_2_, CH_4_ and soil N_2_O, respectively) and sensitivity analysis 4 (Sa4) was performed as a combination of the above three peat soil emission species to express a worst and best case scenario on peat soil emissions. In Sa1-Sa4, the specific lower and upper values (Table 1) were applied and no other parameters were assumed affected in order to focus on the uncertainty of the specific peat soil emission(s) under consideration. For these sensitivity analyses, ha was used as functional unit. The fifth sensitivity analysis (Sa5) was performed on the yield (lower and upper values in Table 1) and in this case, associated secondary parameters were adjusted according to the yield, i.e. indirect N_2_O and diesel consumption for the RCG cases, in order to view the consequences of not obtaining the desired biomass yield as well as obtaining higher yields than assumed. For the latter sensitivity analysis, two alternative functional units were applied, namely kg harvested DM and kg harvested CP.

## 3 Results

**Fig. 3:**
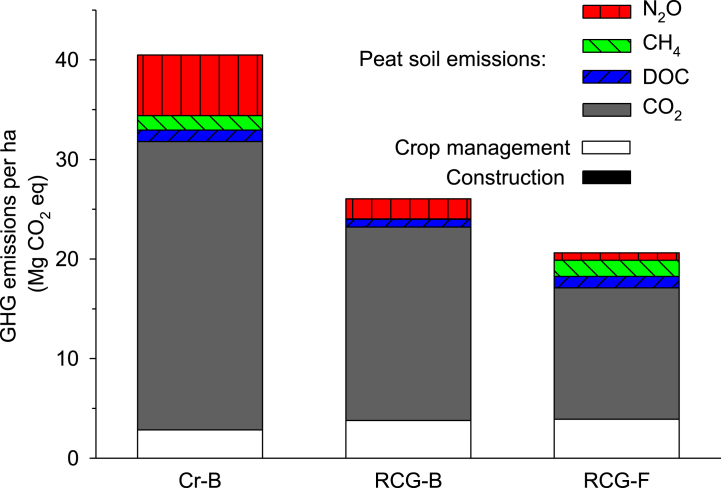
GHG emissions per ha per year for the cases: Present crop production on deep-drained bog (CR-B), and reed canary grass production on rewetted bog (RCG-B) and poorly drained fen (RCG-F). GHG emissions are divided into six categories: Construction work (only relevant for the RCG-B case), crop management (including transportation and field emissions (Table 1)), and the peat soil related/derived emissions, i.e. CO_2_, DOC, CH_4_ and N_2_O. All GHGs are converted to CO_2_eq using global warming factors of 25 for CH_4_ and 298 for N_2_O. Note that the emissions related to construction work for water level rise are too small for visibility in the figure.

The Cr–B case representing the current cultivation management in the raised bog shows the highest GHG emissions per ha (40.5 Mg CO_2_eq ha^−1^ yr^−1^) (Fig. 3). Corresponding values for the RCG-B and RCG-F cases are 26.1 and 20.6 Mg CO_2_eq ha^−1^. Emissions related to crop management are relatively small and represent 7, 14 and 19% of total emissions for the three cases (Cr–B, RCG-B, and RCG-F). Emissions related to rewetting construction in the RCG-B case are negligible (51 kg CO_2_eq ha^−1^ yr^−1^). Focusing on the peat soil GHG emissions, CO_2_ dominates summed emissions from all cases, namely 78, 72 and 62% for Cr–B, RCG-B and RCG-F, respectively, whereas CH_4_ and N_2_O constituted 4, 0 and 8%, and 15, 8 and 4%, respectively, for the three cases (Fig. 3). CH_4_ emissions mainly arise from ditches; this is why the two open-drained cases (Cr–B and RCG-F) show approximately the same level whereas the ditch-free RCG-B case hardly has any CH_4_ emissions.

**Fig. 4:**
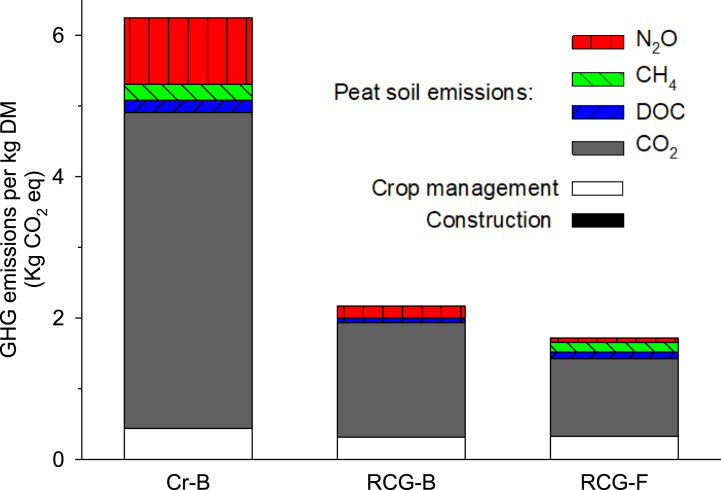
GHG emissions per kg harvested biomass (DM) for each of the three cases: Present crop production on deep-drained bog (CR-B), and reed canary grass production on rewetted bog (RCG-B) and poorly drained fen (RCG-F). GHG emissions are divided into six categories: Construction work (only relevant for the RCG-B case), crop management (including transportation and field emissions (Table 1)), and the peat soil related/derived emissions, i.e. CO_2_, DOC, CH_4_ and N_2_O. All GHGs are converted to CO_2_eq using global warming factors of 25 for CH_4_ and 298 for N_2_O. Note that the emissions related to construction work for water level rise are too small for visibility in the figure.

**Fig. 5:**
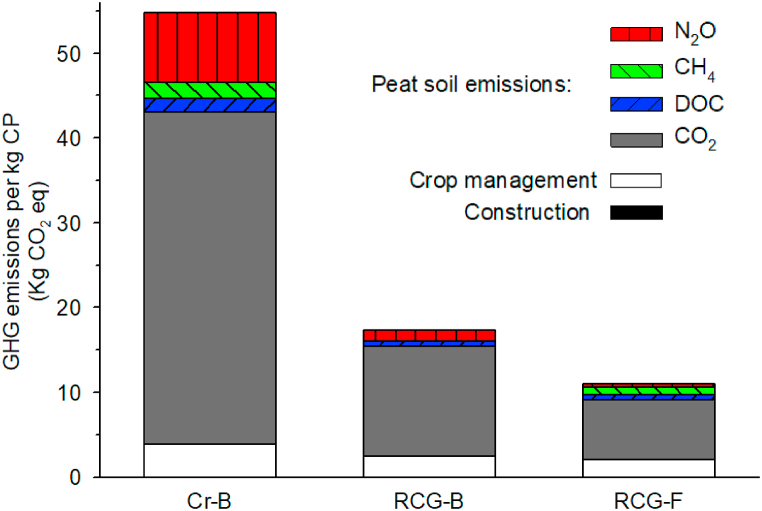
GHG emissions as CO_2_eq per kg harvested crude protein for each of the three cases: Present crop production on deep-drained bog (CR-B) and reed canary grass production on rewetted bog (RCG-B) and poorly drained fen (RCG-F). GHG emissions are divided into six categories: Construction work (only relevant for the RCG-B case), crop management (including transportation and field emissions (Table 1)), and the peat soil related/derived emissions, i.e. CO_2_, DOC, CH_4_ and N_2_O. All GHGs are converted to CO_2_eq using global warming factors of 25 for CH_4_ and 298 for N_2_O. Note that the emissions related to construction work for water level rise are too small for visibility in the figure.

Applying the functional unit 1 kg harvested DM and 1 kg harvested CP, the three cases resulted in 6.2, 2.2, and 1.7; and 54.8, 17.4, and 11.0 kg CO_2_eq kg^−1^, respectively (Fig. 4, Fig. 5).

The sensitivity analyses conducted per ha revealed that the span in the Sa4 (peat soil combined) GHG emissions for the Cr–B, RCG-B and RCG-F cases ranged from 32.0 to 49.4, 19.1 to 33.2 and 12.2–29.1 Mg CO_2_eq ha^−1^ (Table 2), which was clearly dominated by CO_2_ emissions from soil degradation.

**Table 2:** Sensitivity analyses results obtained by replacing the main value with the lower and upper values for each of the peat soil GHG emissions (i.e. CO_2_, CH_4_ and N_2_O) and yields, respectively, while the other values are kept as in the main analysis. In Sa5 regarding yields, the associated values for diesel and indirect N_2_O are also changed according to Table 1. Peat soil combined (Sa4) emerges by combining the lower and upper values, respectively, for each of the peat soil GHG emission species. For analyses Sa1, Sa2, Sa3 and Sa4, 1 ha was used as functional unit, and for Sa5 two alternative functional units were applied, namely 1 kg harvested DM and 1 kg harvested CP.

Case	Cr–B (kg CO_2_eq)<			RCG-B (kg CO_2_eq)<			RCG-F (kg CO_2_eq)<		
**Emission**	**Main**	Lower	Upper	**Main**	Lower	Upper	**Main**	Lower	Upper
Sa1; peat soil CO₂ (per ha)	40,483	35,350	45,983	26,149	20,282	32,016	20,635	14,035	27,235
Sa2; peat soil CH₄ (per ha)	–	39,375	41,590	–	26,122	26,177	–	19,285	21,988
Sa3; peat soil N₂O (per ha)	–	38,235	42,824	–	25,025	27,320	–	20,148	21,150
Sa4; peat soil combined (per ha)	–	31,994	49,432	–	19,131	33,214	–	12,198	29,103
Sa5; yield									
(per kg DM)	6.2	7.4	5.4	2.2	3.3	1.6	1.7	2.6	1.3
(per kg CP)	54.8	64.6	47.6	17.4	26.4	13.0	11.0	16.8	8.1

## 4 Discussion

### 4.1 Comparison of agricultural peatland system and paludiculture peatland systems on bog and fen

The three cases were investigated by applying the most relevant IPCC GHG EFs according to management, nutrient status, drainage and WTD conditions.

The rewetted (undrained) bog case (RCG-B) shows reduced GHG emissions by 35% (14.3 Mg CO_2_eq ha^−1^ yr^−1^) as compared to the drained crop-farm system (Cr–B), although minor overlap is evident in the sensitivity analysis. Sensitivity analyses revealed that only in the Sa4, the GHG emissions overlapped, indicating some uncertainty on the difference between the two cases, however, only present when all peat soil emissions were in the extreme upper part of the interval in the RCG-B case combined with extreme lower values in the Cr–B case. Applying the functional unit of 1 kg biomass DM, GHG emissions were reduced by 65% by changing the bog management from Cr–B to RCG-B, whereas applying the 1 kg CP functional unit resulted in GHG reduction of 68%. It should be noted that rewetting projects in theory could result in rewetted peatlands with water filled ditches and thus such cases would have GHG contributions from CH_4_, which is excluded from the RCG-B case, since in this case the rewetting construction work fills up all ditches. The GHG mitigation from rewetting of peat soils is also concluded by Beetz et al. (2013) [42]; who analyzed the effect of rewetting an intensively managed and fertilized drained grassland on a temperate raised bog. WTD in the intensively managed grassland was −59 cm across the two-year measuring campaign, whereas the extensively managed and unfertilized rewetted site had WTD of approximately −30 cm and was found to have a total global warming potential (GWP) associated to peat soil emissions of around zero. This is considerably lower than our findings, which could be explained by the difference in WTD.

Comparison of the RCG cases shows 21% less GHG emissions for the fen as compared to the bog (5.5 Mg CO_2_eq ha^−1^ yr^−1^), which is caused by the failure of the bog rewetting to increase the water table further than approximately −38 cm. Thus none of the two cases fall into the “rewetted” category for peat soils, since the fen is actually drained, although sparsely, and the bog has a too deep WTD. The nutrient status (rich and poor) differs between the two cases, which results in differentiated EFs. Generally, rewetted nutrient rich sites show larger GWP as compared to nutrient poor as a result of higher emissions of both CO_2_ and CH_4_ because of enhanced peat degradation [6,21]. Thus, the nutrient status itself entails lower emissions in favor of the nutrient poor bog site. If the rewetting event treated in this study would qualify the RCG-B case as rewetted (WTD no deeper than −30 cm) as applied in IPCC et al. (2014) [21] the peat soil CO_2_ EF would become negative, and despite that it would also alter N_2_O and CH_4_ emission factors, a summed reduction as compared to the presented result of approximate 19 Mg CO_2_eq ha^−1^ yr^−1^ would be achieved (to approximate 7 Mg CO_2_eq ha^−1^ yr^−1^; data not shown) and even larger when applying the updated EFs in Wilson et al. (2016) [6]. A study evaluating paludiculture in German fen peatlands, concluded that site emissions ranged from 0 to 8 Mg CO_2_eq ha^−1^, which is considerably less than in this study [43]. Although N_2_O was not considered in the study, the generally lower site emissions were probably a result of wetter site conditions than we assume, and points at that it needs special consideration when fen sites are shallow-drained in the starting point, which might prevent obtaining the desired WTD. Karki et al. (2019) [44] measured and modelled annual CO_2_–C emissions from the soil in a Danish fen with WTD treatments ranging from −1 to −9 cm to be 0.6–2.4 Mg C ha^−1^. This is in line with the above German study and less than the 3.6 Mg C assumed in the present study but also with minor WTD as compared to −13 cm in the shallow drained fen case (RCG-F).

Sensitivity analyses showed overlap emissions between the RCG-B and RCG-F cases on peat soil CO_2_ emissions (Sa1) and combined peat soil emissions (Sa4), respectively, whereas uncertainties on soil CH_4_ and N_2_O were not large enough to make sensitivity intervals overlap. Applying the functional unit of 1 kg biomass DM and CP revealed that GHG emission of RCG-F was, respectively, 23 and 37% below the emission of RCG-B. Due to a substantially overlap in the sensitivity analysis, especially regarding DM as functional unit, more data on actual achieved yields in the RCG-B case is needed in order to confirm the difference between cases. In addition, generally there is a need of more measured GHG fluxes from a range of sites with different nutrient status and WTD in order to allow for further disaggregation of EFs on measured WTD and nutrient status, land-use type, etc. In this study, the WTD of the rewetted bog case (RCG-B) did not reach the −30 cm threshold for rewetted sites as described in IPCC et al. (2014) [21], which point at another important issue, namely that natural hydrology might be difficult to reestablish on bog peat soils situated above the surrounding water bodies, without recovering the sphagnum growth, which has proved difficult without sufficient water supply [45].

A study investigating the consequence of converting drained peat land under cropping management to seasonally-inundated extensively grazed grassland on fen peat soil, reported an 80% reduction in net annual C losses and pointed at transitions from deep-drained to shallow drained grasslands as one of the most efficient means of reducing GHG emissions from peatlands in regions relying on food production [46]. The main reason for this is the reduced level of peat soil degradation when the water level is raised and oxygen supply depressed. The extensively grazed peat soil described in Peacock et al. (2019) [46] had a network of ditches used for water level management rather than buried drain tubes and thus were comparable to the RCG-F case in this study, although an average annual WTD of −51 cm across two study years was reported, which is considerable deeper than the −13 cm WTD in the RCG-F case. This further points at that difficulties in obtaining WTD of −30 cm or closer to the surface might not be isolated to raised bog sites.

Although differences between the cases in our study occur as a direct consequence of the applied emissions factors for each case, we consider the CO_2_ mitigation from rewetting as evident, due to the simple biological explanation behind, in terms of less oxygen for peat degradation, which is supported by multiple studies [1,6,10].

We assumed that the GHG emissions from the rewetted former drained agricultural land (RCG-B) would adhere to emissions associated to undrained lands from day one without any transient period, which is in line with IPCC [21], although this is probably simplified [2,47,48]. Further knowledge on this matter would increase accuracy of the time perspective needed to obtain the calculated GHG mitigation from rewetting events. In addition, more documentation is needed for GHG EFs currently used for wet agricultural production and paludiculture. For instance, effects of the level of nutrient availability for continued crop production (paludiculture) and effects of biomass harvest on peat formation and GHG emission have not been documented thoroughly [49].

### 4.2 Cultivation and construction work emissions

Emissions associated to area management in terms of cultivation practices in this study were 2840, 3900 and 3730 kg CO_2_eq ha^−1^ yr^−1^ for the Cr–B, RCG-B and RCG-F cases, respectively, constituting 7, 14 and 19% of total emissions in the three cases. The larger emissions in the RCG cases reflect the diesel consumption mainly related to the repeated grass-harvest under wet conditions and the upstream GHG emissions related to N fertilizer production.

The relatively low contribution from cultivation points at that abandoning the two peatland sites in this study would only decrease total GHG emissions minor as compared to the RCG cases, although other conditions might increase the difference between paludiculture and abandoned peatlands in terms of e.g. removal of harvested material vs. not removing any biomass from the site. However, even if the sites were left as natural sites, they would still adhere to their respective categories in the IPCC EF system, since the fen site would still be shallow-drained and the bog site would still not adhere to the WTD threshold of −30 cm to be included as *rewetted*.

The tentative assumptions for GHG emissions associated to the construction work needed for rewetting of the bog (RCG-B) was 51 kg CO_2_eq ha^−1^ yr^−1^ when divided on 20 years, revealing a rather negligible contribution to total GHG emissions. The immediate conclusion from this is that construction works that entail decreased WTD in bog peat land rewetting projects, could be beneficial and should not be omitted solely on the argument of large amounts of fuel consumption, but rather be viewed in the full project perspective including economic considerations.

### 4.3 Applying the Danish Tier 2 values for deep-drained peat soil CO_2_ emissions

Since the Danish Tier 2 emission factors for deep-drained agricultural peat soils are currently used for the national reporting to the United Nations Framework Convention on Climate Change [22], an alternative analysis applying the Tier 2 value was performed. The Danish Tier 2 EF (11.5 Mg CO_2_–C ha^−1^ yr^−1^) for peat soil CO_2_ was derived from a national survey based on eight annual fluxes obtained from biweekly GHG measurements of different agricultural managements/sites during 2008–09 [30], and is higher than the corresponding IPCC default value of 7.9 Mg CO_2_–C ha^−1^ yr^−1^ [21]. When applying the Danish Tier 2, the summed GHG emissions from the Cr–B case reaches 54.5 Mg CO_2_eq ha^−1^ yr^−1^ (Fig. A1 and Table A8). There are no Danish Tier 2 for undrained and shallow drained peat soils and thus these results do not change. The RCG-B case then entails a reduction of 52% (28.2 Mg CO_2_eq ha^−1^ yr^−1^), with no overlapping extremes in any sensitivity analyses. Further, the GHG emissions reach 8.5 and 73.7 kg CO_2_eq per kg DM and per kg CP, respectively (Fig. A2 and Fig A3). The increased emissions from the Cr–B case naturally increase the potential GHG reduction as a consequence of the drainage blocking (RCG-B case) and even pass the estimated reduction potential from successful rewetting of cropland of 25.61 Mg CO_2_eq ha^−1^ yr^−1^ as reported as updated emission reduction in Wilson et al. (2016) [6].

### 4.4 Substitution of harvested biomass C

Despite the focus on peat soil emissions in this study, the cases also have a substantial biomass production. This biomass will eventually release the C content in terms of CO_2_ and therefore it is fair to neglect this in the analysis. However, if the harvested C could be either stored or offset other emissions, e.g. fossil fuel, emission intensive products or reduce deforestation, then it could be included in a specific LCA of a rewetting project [26]. Such an approach needs to be very specific on the use of potential biomass harvest, since in theory there are a lot of possible utilizations of the biomass, not just from the production of e.g. RCG on rewetted sites but also of the products cultivated in the deep-drained agricultural systems. RCG has proved suitable in biogas production [18] and in biorefinery protein extraction [17] and it could also serve as feedstock in pyrolysis production aiming for fuel production and biochar for C storage. The harvested biomass C makes out a relatively large proportion of total GHG emissions in the RCG cases, due to higher DM yield and the full-crop harvest of RCG, as compared to the crop farm case, which is also reflected when applying the functional units of kg DM and kg CP. Thus, the specific substitution of the exported C also potentially becomes more important for these cases, which underpin the importance of the purpose for the harvested biomass. If, for instance, the biomass is used for combustion to produce electricity, the GHG load of the replaced grid electricity (e.g. produced from coal vs. wind power) heavily influences the size of the negative emissions achieved from the replacement that can be included in the analysis [50]. Thus the biomass end use compellingly influences the sustainability of the persuasive GHG mitigation of agricultural activities on wet peatlands as compared to drained crop farming. In this context it would be fair to mention that the harvested biomass from the deep-drained case (Cr–B) are aimed (and appropriate) for human consumption, which also needs consideration in regions where food security is challenged, whereas the RCG so far are best suited as animal protein source via green biorefining or to offset fossil fuel.

## 5 Conclusion

Considerable GHG reductions were found when using an LCA approach to investigate consequences of ceasing active drainage of a bog site currently used for potato production by applying IPCC EFs, although the sensitivity analysis revealed minor overlap in extreme ranges of soil emissions.

The nutrient rich and wet fen site performed better in a climate perspective than the non-drained nutrient poor bog site although disaggregated EFs for nutrient rich and poor peat soil generally favors nutrient poor sites. This was mainly due to differences in assumed WTD. Improved and more detailed disaggregated EFs for both poor and rich peat sites according to WTD are needed to shed further light on the possible obtainable GHG reduction from rewetting projects.

Applying the functional units of kg DM and kg CP, rather than area-based functional unit, showed increased reduction potential due to the higher biomass and crude protein yield in RCG grass as compared to cash crops in the deep drained case. Achieving large biomass yields on rewetted or non-/shallow-drained peat soils may also substitute fossil energy or feed production in other areas if the biomass is used for biogas, pyrolysis or in future biorefineries, and this could tip the balance further towards paludiculture or alike cases. Importantly, this could also improve economic feasibility of these management regimes potentially allowing rewetting of more drained peatland or with increased pace.

## Author contribution statement

Henrik Thers: Conceived and designed the experiments; Analyzed and interpreted the data; Wrote the paper.

Marie Trydeman Knudsen: Conceived and designed the experiments; Contributed reagents, materials, analysis tools or data.

Poul Erik Lærke: Conceived and designed the experiments; Performed the experiments; Wrote the paper.

## Data availability statement

Data will be made available on request.

## Declaration of competing interest

The authors declare that they have no known competing financial interests or personal relationships that could have appeared to influence the work reported in this paper
