# Supplementary material for: Comparison of GHG emissions from annual crops in rotation on drained temperate agricultural peatland with production of reed canary grass in paludiculture using an LCA approach
Source: Heliyon. 2023 Jun 15;9(6):e17320. doi: 10.1016/j.heliyon.2023.e17320 (PMC10333457; doi:10.1016/j.heliyon.2023.e17320)
Supplement: Multimedia component 1 [file mmc1.docx]

**Supplementary material for:**
***Comparison of GHG emissions from annual crops in rotation on drained temperate agricultural peatland with production of reed canary grass in paludiculture using an LCA approach***

**Heliyon**

*Henrik Thers, Marie Trydeman Knudsen, Poul Erik Lærke*

Annex A. I. Detailed data for calculations in main paper Page 2

Annex A. II. Applying the Danish tier 2 emission factor on peat soil CO_2_ Page 5

**Annex A. I. Detailed data for calculations in main paper**

Table A1. Details on the summed methane emissions from field and drainage in the Cr-B case. For the conversion from Drainage to Drainage per field ha, the default value of 5% drain surface per ha was applied (IPCC 2014).

|  | **Main (kg CH_4_-C ha^-1^ yr^-1^)** | **Lower (kg CH_4_-C ha^-1^ yr^-1^)** | **Upper (kg CH_4_-C ha^-1^ yr^-1^)** |
| --- | --- | --- | --- |
| Field | 0 | -2.1 | 2.1 |
| Drainage | 873.75 | 251.25 | 1496.25 |
| Drainage per field ha | 43.69 | 12.56 | 74.81 |
| *Total* | *43.69* | *10.46* | *76.91* |

Table A2. Details on the summed methane emissions from field and drainage in the RCG-F case. For the conversion from Drainage to Drainage per field ha, the default value of 5% drain surface per ha was applied (IPCC 2014).

|  | **Main (kg CH_4_-C ha^-1^ yr^-1^)** | **Lower (kg CH_4_-C ha^-1^ yr^-1^)** | **Upper (kg CH_4_-C ha^-1^ yr^-1^)** |
| --- | --- | --- | --- |
| Field | 29.25 | -2.18 | 60.75 |
| Drainage | 395.25 | 213.75 | 576.75 |
| Drainage per field ha | 19.76 | 10.68 | 28.84 |
| *Total* | *49.01* | *8.5* | *89.59* |

Table A3. Detailed information on the individual crops in the Cr-B case, from which the average in Table 1 is derived.

| Crop | **Yield DM (kg ha^-1^)** | **Yield CP (kg ha^-1^)** | **Diesel  (l ha^-1^)** | **N  (kg ha^-1^)** | **P  (kg ha^-1^)** | **K  (kg ha^-1^)** |
| --- | --- | --- | --- | --- | --- | --- |
| Potato | 9160 | 882 | 144 | 75 | 23 | 205 |
| Spring barley | 5780 | 697 | 41 | 113 | 22 | 40 |
| Oat | 4505 | 636 | 41 | 92 | 22 | 40 |

Table A4. N input, output and balance as well as calculation of indirect N_2_O for the three crops in the Cr-B case. All values are per ha per year.

|  |  | **Potato** |  |  |  | **Barley** |  |  |  | **Oat** |  |
| --- | --- | --- | --- | --- | --- | --- | --- | --- | --- | --- | --- |
|  | **main** | lower | upper |  | **main** | lower | upper |  | **main** | lower | upper |
| CO_2_-C degradation (Mg C) | 7.9 |  |  |  | 7.9 |  |  |  | 7.9 |  |  |
| CH_4_-C released (kg C) | 43.69 |  |  |  | 43.69 |  |  |  | 43.69 |  |  |
| N mineralized (kg N) | 300 | 300 | 300 |  | 300 | 300 | 300 |  | 300 | 300 | 300 |
| N fertilizer (kg N) | 75 | 75 | 75 |  | 112.5 | 112.5 | 112.5 |  | 91.5 | 91.5 | 91.5 |
| N deposit (kg N) | 14 |  |  |  | 14 |  |  |  | 14 |  |  |
| Total input (kg N) | 389 | 389 | 389 |  | 427 | 427 | 427 |  | 406 | 406 | 406 |
|  |  |  |  |  |  |  |  |  |  |  |  |
| Harvested N (kg N) | 141.1 | 119.9 | 162.2 |  | 111.5 | 94.8 | 128.2 |  | 101.8 | 86.5 | 117.0 |
| N turnover, residue (kg N) | 77 | 65 | 88 |  | 92 | 78 | 106 |  | 65 | 55 | 74 |
| N turnover, cov. crop (kg N) | 28 | 28 | 28 |  | 28 | 28 | 28 |  | 28 | 28 | 28 |
| N_2_O-N, soil (kg N) | 13 |  |  |  | 13 |  |  |  | 13 |  |  |
| N_2_O-N, direct (kg N) | 1.79 | 1.68 | 1.91 |  | 2.32 | 2.18 | 2.46 |  | 1.84 | 1.74 | 1.93 |
| N_2_O-N, sum (kg N) | 14.8 | 14.7 | 14.9 |  | 15.3 | 15.2 | 15.5 |  | 14.8 | 14.7 | 14.9 |
| N_2_-N (kg N) | 133.1 | 132.1 | 134.2 |  | 137.9 | 136.6 | 139.1 |  | 133.5 | 132.7 | 134.4 |
| NH_3_, fertilizer (kg N) | 3.75 | 3.75 | 3.75 |  | 5.6 | 5.6 | 5.6 |  | 4.6 | 4.6 | 4.6 |
| NH_3_, crops (kg N) | 2.0 | 2.0 | 2.0 |  | 2.0 | 2.0 | 2.0 |  | 2.0 | 2.0 | 2.0 |
| Total gas loss (kg N) | 153.7 | 152.5 | 154.8 |  | 160.8 | 159.4 | 162.2 |  | 154.9 | 154.0 | 155.9 |
| DOC (Mg C) | 0.31 |  |  |  | 0.31 |  |  |  | 0.31 |  |  |
| Mineraliz. from DOC (kg N) | 11.7 | 11.7 | 11.7 |  | 11.7 | 11.7 | 11.7 |  | 11.7 | 11.7 | 11.7 |
| Total output (kg N) | 294.7 | 272.4 | 317.1 |  | 272.3 | 254.2 | 290.4 |  | 256.7 | 240.5 | 272.9 |
| Balance (loss; kg N)) | 94.3 | 116.6 | 72.0 |  | 154.2 | 172.3 | 136.1 |  | 148.8 | 165.1 | 132.6 |
| Indirect N_2_O, eva. (kg N) | 0.1 | 0.1 | 0.1 |  | 0.1 | 0.1 | 0.1 |  | 0.1 | 0.1 | 0.1 |
| Indirect N_2_O, leach. (kg N) | 1.0 | 1.3 | 0.8 |  | 1.7 | 1.9 | 1.5 |  | 1.6 | 1.8 | 1.5 |
| Indirect N_2_O, DOC (kg N) | 0.1 | 0.1 | 0.1 |  | 0.1 | 0.1 | 0.1 |  | 0.1 | 0.1 | 0.1 |
| Total indirect N_2_O (kg N) | 1.2 | 1.5 | 1.0 |  | 1.9 | 2.1 | 1.7 |  | 1.8 | 2.0 | 1.7 |

Table A5. N input, output and balance as well as calculation of indirect N_2_O for the RCG-B and RCG-F cases. All values are per ha per year.

|  |  | **RCG-B** |  |  |  | **RCG-F** |  |
| --- | --- | --- | --- | --- | --- | --- | --- |
|  | **main** | lower | upper |  | **main** | lower | upper |
| CO_2_-C degradation (Mg C) | 5.3 |  |  |  | 3.6 |  |  |
| CH_4_-C released (kg C) | 1.35 |  |  |  | 49.01 |  |  |
| N mineralized (kg N) | 200 | 200 | 200 |  | 255 | 255 | 255 |
| N fertilizer (kg N) | 200 | 200 | 200 |  | 200 | 200 | 200 |
| N deposit (kg N) | 14 | 14 | 14 |  | 14 | 14 | 14 |
| Total input (kg N) | 414 | 414 | 414 |  | 469 | 469 | 469 |
|  |  |  |  |  |  |  |  |
| Harvested N (kg N) | 240 | 160 | 320 |  | 300 | 200 | 400 |
| N_2_O-N, soil (kg N) | 4.3 |  |  |  | 1.6 |  |  |
| N_2_O-N, direct (kg N) | 2.00 | 2.00 | 2.00 |  | 2.00 | 2.00 | 2.00 |
| N_2_O-N, sum (kg N) | 6.30 | 6.30 | 6.30 |  | 3.60 | 3.60 | 3.60 |
| N_2_-N (kg N) | 56.7 | 56.7 | 56.7 |  | 32.4 | 32.4 | 32.4 |
| NH_3_, fertilizer (kg N) | 10.0 | 10.0 | 10.0 |  | 10.0 | 10.0 | 10.0 |
| NH_3_, crops (kg N) | 0.5 | 0.5 | 0.5 |  | 0.5 | 0.5 | 0.5 |
| Total gas loss (kg N) | 73.5 | 73.5 | 73.5 |  | 46.5 | 46.5 | 46.5 |
| DOC (Mg C) | 0.24 |  |  |  | 0.31 |  |  |
| Mineraliz. from DOC (kg N) | 9.1 | 9.1 | 9.1 |  | 21.7 | 21.7 | 21.7 |
| Total output (kg N) | 314 | 234 | 394 |  | 347 | 247 | 447 |
| Balance (loss; kg N)) | 100.7 | 180.7 | 20.7 |  | 122.7 | 222.7 | 22.7 |
| Indirect N_2_O, eva. (kg N) | 0.1 | 0.1 | 0.1 |  | 0.11 | 0.11 | 0.11 |
| Indirect N_2_O, leach. (kg N) | 1.1 | 2.0 | 0.2 |  | 1.3 | 2.4 | 0.2 |
| Indirect N_2_O, DOC (kg N) | 0.1 | 0.1 | 0.1 |  | 0.2 | 0.2 | 0.2 |
| Total indirect N_2_O (kg N) | 1.31 | 2.19 | 0.43 |  | 1.69 | 2.79 | 0.59 |

**Annex A. II. Applying the Danish tier 2 emission factor on peat soil CO_2_**

*Table A6. Inventory table for GHG emissions, yields and input used for calculations of total carbon footprint and sensitivity analyses of the three cases investigated applying the Danish tier 2 emission factor for peat soil CO_2_* (Nielsen et al. 2021)*. Main indicates the values applied in the main analysis and lower and upper values are applied in the sensitivity analysis of uncertainty on specific parameters. Lower and upper values for diesel as well as direct and indirect N_2_O are associated to the sensitivity analyses on yields. The only difference to Table 1 in the main paper is the peat soil CO_2_ emission factor and (caused by that) the indirect N_2_O emission in the Cr-B case. All values are per ha per year.*

| **Case** |  | **Cr-B** | | |  | **RCG-B** | | |  | **RCG-F** | | |
| --- | --- | --- | --- | --- | --- | --- | --- | --- | --- | --- | --- | --- |
| **Inventory** |  | **Main** | Lower | Upper |  | **Main** | Lower | Upper |  | **Main** | Lower | Upper |
| ***Peat soil emissions*** |  |  |  |  |  |  |  |  |  |  |  |  |
| CO_2_ (Mg C) |  | 11.5 | 9.5 | 13.5 |  | 5.3 | 3.7 | 6.9 |  | 3.6 | 1.8 | 5.4 |
| DOC (Mg C) |  | 0.31 | - | - |  | 0.31 | - | - |  | 0.31 | - | - |
| CH_4_ (kg C) |  | 43.7 | 10.5 | 76.9 |  | 1.4 | 0.5 | 2.2 |  | 49.0 | 8.5 | 89.6 |
| N_2_O (kg N) |  | 13.0 | 8.2 | 18.0 |  | 4.3 | 1.9 | 6.8 |  | 1.6 | 0.6 | 2.7 |
| ***Field emissions*** |  |  |  |  |  |  |  |  |  |  |  |  |
| N_2_O, direct (kg N) |  | 2.0 | 1.9 | 2.1 |  | 2.0 | 2.0 | 2.0 |  | 2.0 | 2.0 | 2.0 |
| N_2_O, indirect (kg N) |  | 3.1 | 3.4 | 2.9 |  | 1.3 | 2.2 | 0.4 |  | 1.7 | 2.8 | 0.6 |
| ***Input*** |  |  |  |  |  |  |  |  |  |  |  |  |
| Diesel (L) |  | 75.6 | - | - |  | 164 | 112 | 217 |  | 164 | 112 | 217 |
| Lime (kg) |  | 125 |  |  |  | 0 | - | - |  | 0 | - | - |
| Pesticide (active ingredient; kg) |  | 2.5 | - | - |  | 0 | - | - |  | 0 | - | - |
| N (kg) |  | 93 | - | - |  | 200 | - | - |  | 200 | - | - |
| P (kg) |  | 22 | - | - |  | 43 | - | - |  | 43 | - | - |
| K (kg) |  | 95 | - | - |  | 214 | - | - |  | 214 | - | - |
| ***Output*** |  |  |  |  |  |  |  |  |  |  |  |  |
| Yield DM (Mg) |  | 6.49 | 5.51 | 7.45 |  | 12.0 | 8.0^g^ | 16.0 |  | 12.0 | 8.0 | 16.0 |
| Yield N (kg) |  | 118 | 100 | 136 |  | 240 | 160 | 320 |  | 300 | 200 | 400 |

Hyphens indicate that the specific parameter is not relevant for sensitivity analysis.

Table A7. N input, output and balance as well as calculation of indirect N_2_O for the three crops in the Cr-B case applying the Danish Tier 2 emission factor on agricultural peat soil CO_2_. All values are per ha per year.

|  |  | **Potato** |  |  |  | **Barley** |  |  |  | **Oat** |  |
| --- | --- | --- | --- | --- | --- | --- | --- | --- | --- | --- | --- |
|  | **main** | lower | upper |  | **main** | lower | upper |  | **main** | lower | upper |
| CO_2_-C degradation (Mg C) | 11.5 |  |  |  | 11.5 |  |  |  | 11.5 |  |  |
| CH_4_-C released (kg C) | 43.69 |  |  |  | 43.69 |  |  |  | 43.69 |  |  |
| N mineralized (kg N) | 436 | 436 | 436 |  | 436 | 436 | 436 |  | 436 | 436 | 436 |
| N fertilizer (kg N) | 75 | 75 | 75 |  | 112.5 | 112.5 | 112.5 |  | 91.5 | 91.5 | 91.5 |
| N deposit (kg N) | 14 |  |  |  | 14 |  |  |  | 14 |  |  |
| Total input (kg N) | 525 | 525 | 525 |  | 563 | 563 | 563 |  | 542 | 542 | 542 |
|  |  |  |  |  |  |  |  |  |  |  |  |
| Harvested N (kg N) | 141.1 | 119.9 | 162.2 |  | 111.5 | 94.8 | 128.2 |  | 101.8 | 86.5 | 117.0 |
| N turnover, residue (kg N) | 77 | 65 | 88 |  | 92 | 78 | 106 |  | 65 | 55 | 74 |
| N turnover, cov. crop (kg N) | 28 | 28 | 28 |  | 28 | 28 | 28 |  | 28 | 28 | 28 |
| N_2_O-N, soil (kg N) | 13 |  |  |  | 13 |  |  |  | 13 |  |  |
| N_2_O-N, direct (kg N) | 1.79 | 1.68 | 1.91 |  | 2.32 | 2.18 | 2.46 |  | 1.84 | 1.74 | 1.93 |
| N_2_O-N, sum (kg N) | 14.8 | 14.7 | 14.9 |  | 15.3 | 15.2 | 15.5 |  | 14.8 | 14.7 | 14.9 |
| N_2_-N (kg N) | 133.1 | 132.1 | 134.2 |  | 137.9 | 136.6 | 139.1 |  | 133.5 | 132.7 | 134.4 |
| NH_3_, fertilizer (kg N) | 3.75 | 3.75 | 3.75 |  | 5.6 | 5.6 | 5.6 |  | 4.6 | 4.6 | 4.6 |
| NH_3_, crops (kg N) | 2.0 | 2.0 | 2.0 |  | 2.0 | 2.0 | 2.0 |  | 2.0 | 2.0 | 2.0 |
| Total gas loss (kg N) | 153.7 | 152.5 | 154.8 |  | 160.8 | 159.4 | 162.2 |  | 154.9 | 154.0 | 155.9 |
| DOC (Mg C) | 0.31 |  |  |  | 0.31 |  |  |  | 0.31 |  |  |
| Mineraliz. from DOC (kg N) | 11.7 | 11.7 | 11.7 |  | 11.7 | 11.7 | 11.7 |  | 11.7 | 11.7 | 11.7 |
| Total output (kg N) | 294.7 | 272.4 | 317.1 |  | 272.3 | 254.2 | 290.4 |  | 256.7 | 240.5 | 272.9 |
| Balance (loss; kg N)) | 230.3 | 252.6 | 208.0 |  | 290.2 | 308.3 | 272.1 |  | 284.8 | 301.1 | 268.6 |
| Indirect N_2_O, eva. (kg N) | 0.1 | 0.1 | 0.1 |  | 0.1 | 0.1 | 0.1 |  | 0.1 | 0.1 | 0.1 |
| Indirect N_2_O, leach. (kg N) | 2.5 | 2.8 | 2.3 |  | 3.2 | 3.4 | 3.0 |  | 3.1 | 3.3 | 3.0 |
| Indirect N_2_O, DOC (kg N) | 0.1 | 0.1 | 0.1 |  | 0.1 | 0.1 | 0.1 |  | 0.1 | 0.1 | 0.1 |
| Total indirect N_2_O (kg N) | 2.7 | 3.0 | 2.5 |  | 3.4 | 3.6 | 3.2 |  | 3.3 | 3.5 | 3.1 |

Fig. A1. GHG emissions per ha for each case applying the Danish Tier 2 for peat soil CO_2_ emissions in the Cr-B case. GHG emissions are divided into six categories: Construction work (only relevant for the RCG-B case), crop management (including transportation and field emissions (Table 1)), and the peat soil related/derived emissions, i.e. CO_2_, DOC, CH_4_ and N_2_O. All GHGs are converted to CO_2_eq using global warming factors of 25 for CH_4_ and 298 for N_2_O. Note that the emissions related to construction work for water level rise are too small for visibility in the figure.

Fig. A2. GHG emissions per kg harvested biomass (DM) for each case applying the Danish Tier 2 for peat soil CO_2_ emissions in the Cr-B case. GHG emissions are divided into six categories: Construction work (only relevant for the RCG-B case), crop management (including transportation and field emissions (Table 1)), and the peat soil related/derived emissions, i.e. CO_2_, DOC, CH_4_ and N_2_O. All GHGs are converted to CO_2_eq using global warming factors of 25 for CH_4_ and 298 for N_2_O. Note that the emissions related to construction work for water level rise are too small for visibility in the figure.

Fig. A3. GHG emissions per kg harvested crude protein (CP) for each case applying the Danish Tier 2 for peat soil CO_2_ emissions in the Cr-B case. GHG emissions are divided into six categories: Construction work (only relevant for the RCG-B case), crop management (including transportation and field emissions (Table 1)), and the peat soil related/derived emissions, i.e. CO_2_, DOC, CH_4_ and N_2_O. All GHGs are converted to CO_2_eq using global warming factors of 25 for CH_4_ and 298 for N_2_O. Note that the emissions related to construction work for water level rise are too small for visibility in the figure.

*Table A8. Sensitivity analyses results when applying the Danish tier 2 emission factor on peat soil CO_2_. Results are obtained by replacing the main value with the lower and upper value for each of the peat soil GHG emissions (i.e. CO_2_, CH_4_ and N_2_O) and yields, respectively, while the other values are kept as in the main analysis. In Sa5 regarding yields, the associated values for diesel and indirect N_2_O are changed correspondingly according to Table 1. Peat soil combined (Sa4) emerges by combining the lower and upper values, respectively, for each of the peat soil GHG species. For analyses Sa1, Sa2, Sa3 and Sa4, ha was used as functional unit, and for Sa5 two alternative functional units were applied, namely kg harvested DM and kg harvested CP.*

| **Case** | **Cr-B**  **(kg CO_2_eq)** | | |  | **RCG-B**  **(kg CO_2_eq)** | | |  | **RCG-F**  **(kg CO_2_eq)** | | |
| --- | --- | --- | --- | --- | --- | --- | --- | --- | --- | --- | --- |
| **Emission** | **Main** | Lower | Upper |  | **Main** | Lower | Upper |  | **Main** | Lower | Upper |
| Sa1; peat soil CO₂  (per ha) | 54385 | 47052 | 61719 |  | 26149 | 20282 | 32016 |  | 20635 | 14035 | 27235 |
| Sa2; peat soil CH₄  (per ha) | - | 53278 | 55493 |  | - | 26122 | 26177 |  | - | 19285 | 21988 |
| Sa3; peat soil N₂O  (per ha) | - | 52138 | 56727 |  | - | 25025 | 27320 |  | - | 20148 | 21150 |
| Sa4; peat soil combined (per ha) | - | 43697 | 65168 |  | - | 19131 | 33214 |  | - | 12198 | 29103 |
| Sa5; yield |  |  |  |  |  |  |  |  |  |  |  |
| (per kg DM) | 8.4 | 9.9 | 7.3 |  | 2.2 | 3.3 | 1.6 |  | 1.7 | 2.6 | 1.3 |
| (per kg CP) | 73.7 | 86.7 | 64.0 |  | 17.4 | 26.4 | 13.0 |  | 11.0 | 16.8 | 8.1 |
